# Does altered protein metabolism interfere with postmortem degradation analysis for PMI estimation?

**DOI:** 10.1007/s00414-018-1814-8

**Published:** 2018-03-02

**Authors:** A. Zissler, B. Ehrenfellner, E. E. Foditsch, F. C. Monticelli, S. Pittner

**Affiliations:** 1 Department of Biosciences, Paris-Lodron University of Salzburg, Salzburg, Austria; 2 Department of Forensic Medicine and Forensic Neuropsychiatry, Paris-Lodron University of Salzburg, Salzburg, Austria; 3 Spinal Cord Injury and Tissue Regeneration Center Salzburg, Paracelsus Medical University, Salzburg, Austria

**Keywords:** PMI estimation, Skeletal muscle, Protein, Degradation, Metabolism

## Abstract

An accurate estimation of the postmortem interval (PMI) is a central aspect in forensic routine. Recently, a novel approach based on the analysis of postmortem muscle protein degradation has been proposed. However, a number of questions remain to be answered until sensible application of this method to a broad variety of forensic cases is possible. To evaluate whether altered in vivo protein metabolism interferes with postmortem degradation patterns, we conducted a comparative study. We developed a standardized animal degradation model in rats, and collected additional muscle samples from animals recovering from muscle injury and from rats with developed disuse muscle atrophy after induced spinal cord injury. All samples were analyzed by SDS-PAGE and Western blot, labeling well-characterized muscle proteins. Tropomyosin was found to be stable throughout the investigated PMI and no alterations were detected in regenerating and atrophic muscles. In contrast, significant predictable postmortem changes occurred in desmin and vinculin protein band patterns. While no significant deviations from native patterns were detected in at-death samples of disuse muscle atrophy, interestingly, samples of rats recovering from muscle injury revealed additional desmin and vinculin degradation bands that did not occur in this form in any of the examined postmortem samples regardless of PMI. It remains to be investigated whether in vivo-altered metabolism influences postmortem degradation kinetics or if such muscle samples undergo postmortem degradation in a regular fashion.

## Introduction

To figure out when a person died can have major implications in ongoing criminal investigations but can also play a role in inheritance law and other legal issues. If valid crime scene evidence or trustworthy testimonies are lacking, investigators and forensic experts have to rely on biomedical traces to delimitate the PMI as precisely as possible.

The most commonly used methods to date include the measurement and comparison of body and environmental temperature [1], the examination of so called supravital reactions [2], the evaluation of the progression of rigor mortis [3] or hypostasis [4], and the analysis of necrotrophic insects and their developmental stages in course of forensic entomology [5]. Although these and other methods provide valuable information about the time since death in many cases, all of them come with certain restrictions and temporal limitations. Thus, additional methods are required and extensive research is conducted in this field. Most newly developed approaches, however, did so far not exceed early experimental stages. Recently, a novel approach based on the analysis of postmortem muscle protein degradation has been proposed [6] that has already provided crucial evidence for the progression of events in a first forensic case [7]. However, a number of questions remain to be answered in the context of postmortem protein degradation to be able to sensibly apply this method to a broad variety of forensic cases.

Among those questions is the demand of discriminability of postmortem protein degradation patterns from those of altered in vivo protein metabolism. Protein degradation, although often predominately associated with postmortal processes, is a ubiquitous, complex, evolutionary conserved process in all body cells [8]. Regular protein turnover in muscle fibers is mainly facilitated by the ubiquitin-proteasome pathway and cathepsins of the autophagy-lysosome system [9]. Additionally, caspases are especially known for their activity in programmed cell death such as apoptosis [10] and in necrosis [11]. As the 26S proteasome [12], the autophagy-lysosomal pathway [13], and the catalytic activation of caspases [14] require ATP, which is depleted in postmortem conditions [15], the Ca2+-activated calpains are considered to predominately contribute in postmortem stages [16, 17]. While in living tissue, this system is usually inhibited by calpastatin and low cytoplasmic calcium concentrations [18], it gains importance upon the postmortal increase of intracellular calcium [19]. Postmortem decomposition of skeletal muscle by the calpain system has been described in detail by studies in context of increasing meat tenderness upon storage time and conditions [16].

These decomposition processes are also traced with the above-mentioned approach for PMI estimation. But also enhanced in vivo protein turnover might be detectable (e.g., as additional protein bands) by the applied methodology and could, thus, problematically interfere with PMI estimation.

Occasions with such altered protein metabolism may include cases with preconditions as injury, atrophy, specific diseases, regeneration, and extensive physical training. Although rarely, formation of degradation products as in postmortem decomposition has been reported in in vivo muscle tissue with altered protein metabolism (diaphragm hypoxia [20], heart muscle ischemia [21], and in serum samples from patients with various skeletal muscle disorders [22]). If such protein fragments also occur in in vivo muscle tissue, there must either be a clear possible distinction between in vivo protein fragments and degradation fragments to avoid interference with PMI estimation, or else appropriate exclusion criteria are required for muscle protein analysis in the context of time since death estimation.

To evaluate whether altered in vivo turnover interferes with postmortem degradation patterns, we conducted a comparative study. We therefore developed a standardized animal degradation model in rats, using muscle samples dissected at 0, 1, 2, 3, and 4 days postmortem. We additionally collected muscle samples from animals recovering from a cardiotoxin-induced muscle injury at 2, 4, and 7 days post injury and from rats with developed disuse muscle atrophy 28 days after complete spinal cord injury. All samples were processed according to standard protocols and analyzed by SDS-PAGE and Western blot, labeling the well-characterized muscle proteins desmin, vinculin, and tropomyosin.

## Material and method

### Animal models and study design

To establish a standard degradation model, 20 adult male Sprague Dawley rats were anesthetized with isoflurane and killed by cervical dislocation. Muscle samples (*M. quadriceps femoris*) were immediately dissected after death from four animals. The remaining animals were stored in a climate chamber at a constant temperature setting of 20 °C and a group of four individuals was sampled respectively at 1, 2, 3, and 4 h postmortem (hpm).

To investigate altered antemortem protein turnover in acute injury, 12 adult male Sprague Dawley rats were anesthetized by isoflurane inhalation and received a muscle injury by administration of 500 μL of 10 μM cardiotoxin (CTX from *Naja mossambica mossambica*) in phosphate-buffered saline (PBS) into the left hind limb (*M. quadriceps femoris*). This muscle injury model is commonly used for the induction of a degradation–regeneration cycle in skeletal muscle tissue [23, 24]. On post injury days 2, 4, and 7, four animals, respectively, were anesthetized with isoflurane, killed by cervical dislocation, and thigh muscles were excised.

Additionally, atrophic muscle samples were collected from four female adult Lewis rats that underwent a complete spinal cord transection at the vertebral thoracic level T9. Motor dysfunction and recovery after complete spinal cord transection was assessed using the Basso, Beattie, and Bresnahan locomotor rate scale (BBB) on days 1, 15, and 28 post injury [25]. Twenty-eight days after, injury rats were euthanatized under deep ketamine-xylazine-acepromazine anesthesia and hind limb muscles (*M. quadriceps femoris*) were excised.

All experimental procedures involving live animals were performed in accordance with the animal experiment guidelines issued by the Austrian Federal Ministry of Science, Research, and Economy, which satisfy all international ethical requirements for the use of animals in experimental research studies.

### Sample preparation

Excised muscles were subdivided to smaller pieces (approximately 5 × 5 × 5 mm), snap frozen, and stored in liquid nitrogen. All samples were homogenized by cryogenic grinding and subsequent sonication. 10 × vol/wt RIPA buffer, together with a protease inhibitor cocktail was used as lysis and extraction buffer. The sample solution was centrifuged and the supernatant was used for analysis. The respective protein concentration was determined using BCA-Assay. All samples were diluted to equal overall protein content prior to analysis.

### Electrophoresis and Western blotting

Electrophoresis (SDS-PAGE) was run on 10% polyacrylamide resolving gels and 5% stacking gels, according to our standard protocol [6]. Total protein of 30 μg was prepared, denatured at 90 °C for 5 min, and inserted into the gel wells. Following electrophoresis, the proteins were transferred from the gels onto polyvinylidine fluoride (PVDF) membranes and stored at − 20 °C. For immunolabeling, the membranes were blocked in Tris-buffered saline (TBS) with 1% dried milk as a blocking agent. The following primary antisera were used: mouse monoclonal anti-tropomyosin, mouse monoclonal anti-desmin, and mouse monoclonal anti-vinculin. HRP-conjugated polyclonal goat anti-mouse was applied as secondary antibody. All antibodies were diluted in blocking agent and applied for at least 1 h. After each antibody application, the membranes were extensively washed and rinsed in TBS. Antibody staining was visualized by application of chemiluminescence substrate and documented using a digital gel analysis system.

### Data interpretation and statistics

Protein bands were measured using the gel analysis tools of ImageJ software (1.48 v NIH, National Institutes of Health, USA). Band patterns obtained from the uninjured 0 hpm samples were considered the native form of the protein. All signals on the blot with and intensity of <1% of this native band were regarded background (i.e., not a protein band). This enabled binarization of the results (presence vs. absence of protein bands). Additional bands or the loss of native bands compared to the control samples were considered alterations of the band pattern. Chi-squared tests were used to determine whether there was a significant difference in the observed band frequencies within the different PMI groups (i.e., significant change of the band pattern over time). Significance levels were set at *p* < 0.05.

## Results

### Methodological observation

As expected, there were no significant differences observed on behalf of sampling and sample preparation. A single sample, obtained from a rat with atrophic muscle tissue, yielded a lower overall protein concentration and less volume of supernatant compared to the other samples. This was much rather considered a methodological issue than a systemic (atrophy induced) effect. During sampling, it is impossible to assess the composition of a tissue sample on behalf of the content of fat, connective tissue, vessels, neural tissue, and muscle. This can, in some cases, lead to concentration deviations as the one observed.

### Postmortem degradation

**Fig. 1:**
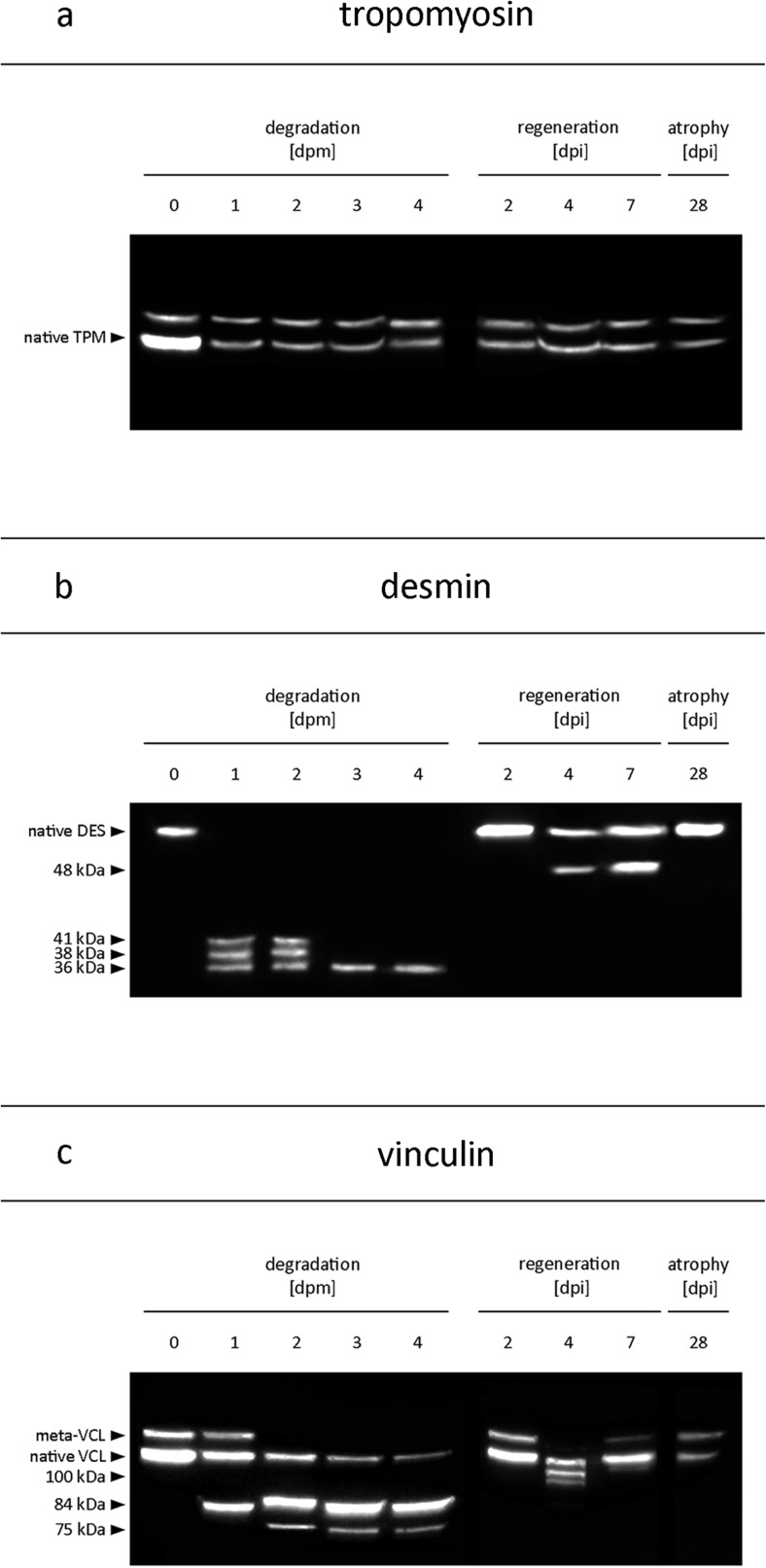
Western blot analyses of proteins and protein fragments in skeletal muscle samples from three different experimental groups. While tropomyosin (TPM) (**a**) depicted no changes of the native protein pattern (i.e., degradation model at 0 days postmortem, dpm), desmin (DES) (**b**) and vinculin (VCL) (**c**) showed distinct alterations, such as the loss of native bands (desmin and meta-vinculin) and the appearance of different protein fragments, with increasing time postmortem, as well as during regeneration from an induced injury at 4 and 7 days post injury (dpi). No alterations were found in samples collected at 2 dpi, or in disuse atrophy samples, collected 28 days (dpi) after complete spinal cord injury. *Black arrow heads* indicate the native bands of the proteins as well as the estimated size of the, respectively, detected protein fragments, by the use of a pre-stained molecular weight protein ladder

**Fig. 2:**
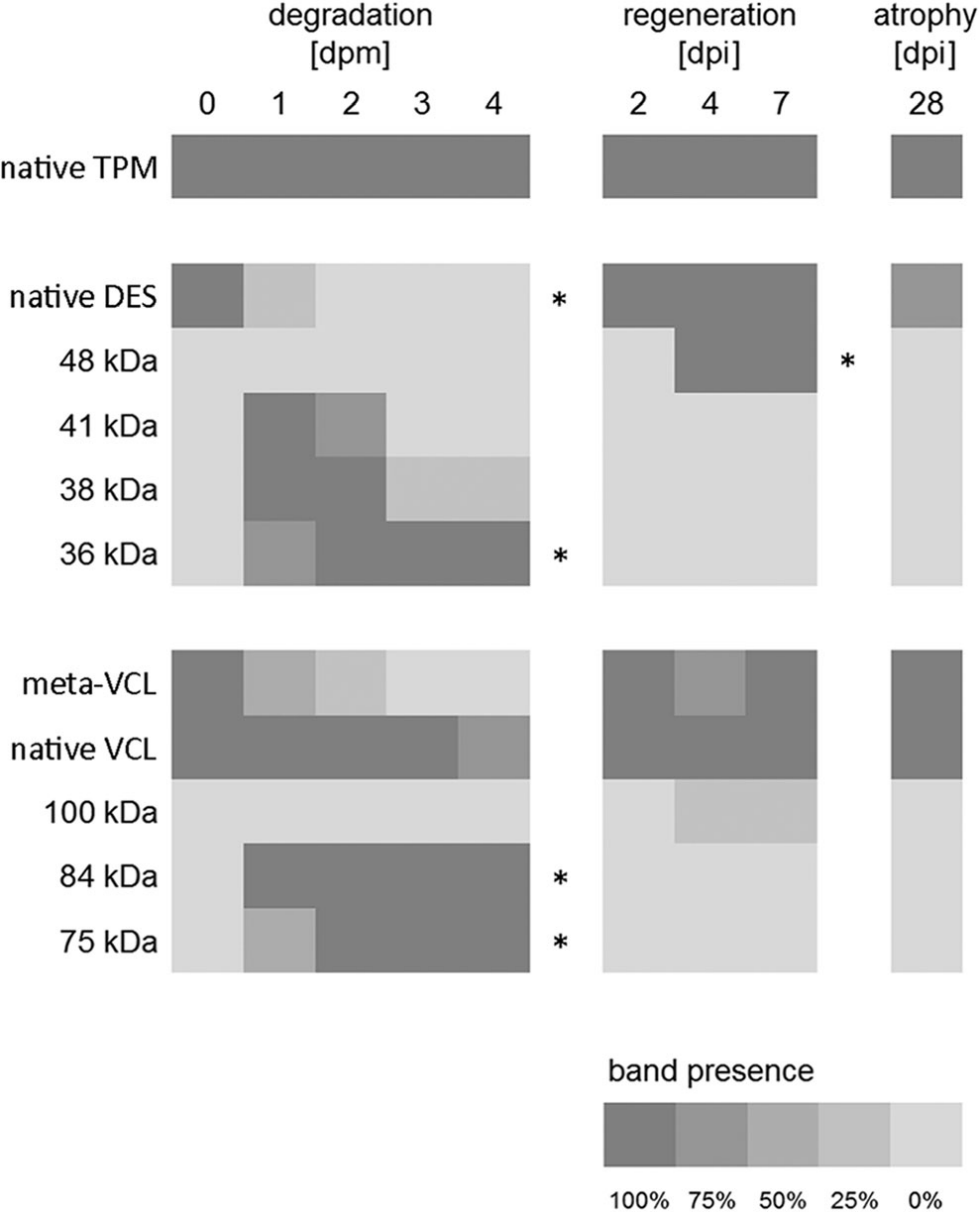
Heat map depicting the frequency of band presence in all tested groups (*n* = 4 each group). Tropomyosin (TPM) bands were detected in all analyzed samples. Frequencies of native bands occasionally decreased (native desmin (DES), vinculin (VCL) and meta-vinculin) and various fragments (indicated by estimated molecular Sweight in kDa) appeared with increasing PMI, and in samples from regenerating muscle. Asterisks indicate significant changes of band patterns, as determined by Chi-squared tests (*p* < 0.05)

Tropomyosin Western blots resulted in characteristic double bands at approximately 36 and 38 kDa, most likely representing two isoforms [26] and no degradation products in all samples regardless of increasing PMI (Figs. 1a and 2). Analysis of this protein was considered a positive control for this experimental series.

Analysis of desmin (Figs. 1b and 2) resulted in a distinct native band at approximately 50 kDa. This band was degraded in three of four samples dissected 1 day postmortem and it was not detectable in any sample with a PMI of 2 days or longer, representing a significant loss of the native desmin band (*p* < 0.05). In addition to this native protein band, characteristic degradation products were detected in all samples with a PMI of 1 day postmortem or longer. One degradation product (41 kDa fragment) occurred transiently at 1 and 2 days postmortem in three and four samples, respectively, and was no longer detectable at 3 and 4 days postmortem. A second degradation product (38 kDa fragment) was present at 1 and at 2 days postmortem in all analyzed samples, and disappeared transiently in samples with a PMI of 3 and 4 days (only detectable in one of the three and four dpi samples respectively). A third degradation product (36 kDa fragment) occurred with significant frequency at approximately 36 kDa (*p* < 0.05). It was detected in three of the four samples dissected 1 day postmortem and in all samples with a longer PMI.

Western blot analysis of vinculin revealed native 117 kDa vinculin bands in most of the investigated samples over the investigated postmortem period (Figs. 1c and 2). Only a single sample, notably sampled from an animal at 4 days postmortem, depicted a lack of this band. An additional band at approximately 135 kDa, commonly referred to as the splice variant meta-vinculin [27], was detected in all at-death samples. This band was identified to be susceptible to postmortem degradation, being detectable in two of the samples dissected 1 day postmortem, in one of the samples dissected 2 days postmortem and in none of the samples dissected 3 and 4 days postmortem. Typical postmortem degradation products were detected in samples with a PMI of 1 day or longer. In all these samples, a band at approximately 84 kDa (84 kDa fragment) was present. A second band at 75 kDa (75 kDa fragment) was detected in only two of the samples dissected 1 day postmortem but in all samples with a longer PMI.

### Potentially altered antemortem protein turnover

Tropomyosin Western blots again resulted in characteristic double bands at 36 and 38 kDa, in all muscle samples of the regeneration cycle and in all samples of muscle atrophy (Figs. 1a and 2). Also in these experimental series, analysis of this protein was regarded a positive control.

The native desmin band was detected in all samples gathered from animals during muscle regeneration and three of the four animals with muscle atrophy (Figs. 1b and 2). Notably, the single sample lacking this band was the one with the low overall protein concentration mentioned above. None of the aforementioned degradation products were detected in any of the samples from animals with muscle injury or atrophy. However, all animals recovering from muscle injury depicted additional bands at approximately 48 kDa on day 4 and day 7 post injury that were not detected in any other samples. This represented a significant alteration (*p* < 0.05) compared to the muscles dissected on day 2 post injury and the muscles of animals used for the protein degradation model.

All injured and atrophic muscles depicted the native vinculin band. Only a single sample from an animal recovering from injury depicted a loss of the meta-vinculin band on day 4 post injury. While none of the rats recovering from injury or with present atrophy showed the smaller 84 and 75 kDa fragments of muscles in the postmortem degradation model (Figs. 1c and 2), an additional band at approximately 100 kDa (100 kDa fragment) was detected in one of the animals of the 4 days and the 7 days post injury group respectively. This 100 kDa fragment was not detected in any muscle sample of the animals used for the protein degradation model. Notably, the mentioned sample of the 4 days post injury group was the one lacking the meta-vinculin band.

## Discussion

With this comparative study, we demonstrate the complexity and yet predictability of protein metabolism under various circumstances including muscle regeneration and atrophy and postmortem degradation. To mimic muscle injury and atrophy, we used common rat injury models including a cardiotoxin-based muscle injury-regeneration model and a spinal cord injury-based atrophy model. Although observations obtained from animals are not necessarily transferable to humans, and thus the ethical legitimacy has to be carefully considered [28, 29], it has been shown that animal models substantially contribute to improve the applicability of medico-legal approaches in crime investigations and to provide an important foundation for basic research [6, 30]. Specifically, the limits and possibilities to compare postmortem protein degradation in animal models and human tissue have been reported [31].

By means of the development of the protein degradation model in rat muscle tissue, we established a valuable comparison model for the present study and for future experiments. Tropomyosin, a structural protein of the myofibrillar complex, was confirmed to be a reliable positive marker within the investigated period of postmortem time and is, furthermore, well applicable as a positive control for the described regeneration and atrophy model. Moreover, the detected significant and predictable postmortem changes in desmin and vinculin band patterns support the reported susceptibility to proteolysis of these structural proteins interconnecting myofibrils and the sarcolemma [6, 32, 33]. The detection of predictable desmin and vinculin degradation profiles in rats confirms ubiquitous degradation kinetics across several mammalian species including mouse, pig, and humans [31], and therefore strengthens the applicability of animal models for research on this PMI estimation approach.

While the main proteolysis pathways in living cells require ATP [12–14], Ca2+-dependent calpains are inhibited and indirectly activated as a consequence of decreasing ATP levels in postmortem tissue [15], conceding calpain cleavage one of the most important autolytic process postmortem [16, 17]. All investigated proteins, indeed, are known substrates of calpains [34]. This might also explain the presence of the distinct desmin and vinculin protein fragments found during the investigated PMI in the present study. Tropomyosin, although a known substrate for calpain cleavage, was found to be stable over the investigated period of 4 days postmortem. Nevertheless, it might undergo degradation in later phases and thus be of interest for PMI estimation in advanced degradation.

Although protein turnover occurs constantly in living tissue, we detected distinct native bands of tropomyosin, desmin, and vinculin in all 0 dpm samples of the rat degradation model. The presence of distinct native protein bands of various proteins in at-death control samples of healthy rats and of different mammalian species including mice [31] and pigs [6, 35] demonstrates that regular protein turn over in antemortem muscle cannot be detected by means of the applied Western blot technique and, thus, does not substantially interfere with protein analysis and the present PMI estimation method. This lack of detection is most likely due to a combination of various factors. For once, regular metabolism, and especially catabolism, does not occur simultaneously in all, or a significant amount of cells and is, thus, beyond a methodological detection limit. And, as long as the ATP-dependent proteolytic systems (predominately 26S proteasome and autophagy) are maintained in living organisms, protein degradation products are further processed to undetectable (by the applied method) smaller units and ultimately single amino acids [36].

It is, however, unclear how altered antemortem metabolism interferes with protein degradation kinetics. In forensic cases, disuse atrophy exemplifies a common phenomenon of altered muscle turnover, in which protein degradation rates exceed protein synthesis [9]. We found no significant deviation from a native state in at-death samples of disuse muscle atrophy. However, a single sample did neither reveal a native desmin band nor any desmin degradation product. Whether this is an actual systemic effect or rather a methodic error remains to be determined. Although an increase in the expression of calpains and cathepsins is demonstrated in muscle unloading, the primary degradation pathway is thought to be the regular ubiquitin-proteasome mechanism [37–39]. Similarly, as in the control muscles of regular protein turnover, a lack of distinct degradation products might be additionally due to a readily disintegration of myofibrillar proteins into small peptides and amino acids. However, it remains unclear and therefore inevitably important to further investigate, whether disuse atrophy influences postmortem degradation kinetics, as the lack of significant difference in in vivo patterns does not necessarily imply a similar and unaffected postmortem degradation sequence.

While there is clear indication that a decrease in protein synthesis occurs in disuse atrophy, the role of protein degradation remains to be clarified [40, 41]. The results of the present study provide additional evidence for a depression in protein synthesis rather than enhanced proteolysis. While disuse muscle atrophy is induced by a variety of common conditions, including starvation, immobilization, unloading and denervation [40, 41], and muscle atrophy, as a cachexia symptom, can also be caused by diseases including cancer, HIV/AIDS, chronic obstructive pulmonary disease (COPD), chronic heart failure, and chronic kidney disease [42]. These forms of muscle atrophy are often associated with the release of inflammatory cytokines, systemic inflammation, and a gross alteration in protein metabolism [42–44]. It therefore remains to be investigated if those forms of disease-related muscle wasting might produce different protein band appearance in at-death muscle samples and/or different protein degradation kinetics.

Such as enhanced protein turnover due to disease, muscle protein metabolism is significantly altered in a muscle injury-regeneration cycle. We found no alterations in samples obtained 2 days post injury. However, interestingly, samples of rats of the day 4 and 7 post injury groups revealed additional desmin degradation bands that did not show up in any of the postmortem samples regardless of PMI. By analyzing vinculin, this phenomenon reoccurred in one of the day 4 and day 7 post injury samples. Degradation of muscle proteins during an injury-regeneration cycle is rarely investigated. However, desmin has been found to be susceptible to proteolysis during injury, with a significant band loss within hours after (similarly) induced muscle injury. No desmin fragmentation was reported during the inflammation and destruction phase of muscle injury in those studies, but desmin reappearance was demonstrated on day 2 post injury [45, 46]. It is exceptional that, in contrast to later regeneration stages, no protein fragments for both proteins were found within the 2 days post injury samples. This provides reason to speculate whether the protein fragments found in later regeneration phases are involved in regeneration processes. It remains to be determined whether the observed protein bands in at-death samples of injured muscle tissue represent actual degradation products, or if the bands represent isoforms of reappearing desmin and vinculin within regenerating fibers. It can also not be excluded that the detected protein patterns could be detected within narrower sampling intervals postmortem (e.g., between 0 and 24 h pm). Regardless of how exactly those additional protein fragments are formed, just as mentioned above, it is required to further examine whether in vivo altered muscle undergoes postmortem degradation in a regular fashion, or if these alterations provide (i) a head-start, (ii) varying kinetics, or even (iii) completely different postmortem degradation patterns. Depending on the findings, the postmortem degradation model will have to be (i) shifted, (ii) a correction factor provided, or (iii) excluded for sensible time since death estimation.

## Conclusion

Estimation of the time since death by analysis of muscle protein degradation is a decent new tool in forensic science. However, to improve the reliability and broaden the applicability, some questions remain to be answered. Enhanced antemortem protein metabolism might alter at-death protein patterns or even postmortem degradation kinetics. Although we found indication for no major interference of enhanced antemortem protein turnover in “zero-hour samples,” it is implied to investigate whether there could be influence on postmortem degradation kinetics. Especially in cases of muscle regeneration with (partly) necrotic tissue, in which there is a deviation of band appearance compared to healthy tissue, it seems worth to further test whether the investigated proteins undergo postmortem degradation in a regular fashion, or whether kinetics are altered. To further establish the new PMI estimation approach and to improve the applicability of the method, possible influencing factors resulting from altered antemortem protein turnover must be considered to be investigated and validated in humans. Other conditions with altered metabolism will also have to be analyzed. It has for example been shown that members of the calpain family are increasingly activated after severe and/or prolonged eccentric exercise and in exercise-induced muscle damage [47, 48]. It therefore remains to be investigated whether enhanced protein metabolism during exercise and phases of extensive physical training (e.g., body building) can influence antemortem metabolic conditions in a way that postmortem degradation kinetics are potentially influenced. Until these uncertainties are answered, we suggest critical cases to be excluded from PMI estimation by analysis of muscle protein degradation.
